# Protective Effects of Two Safflower Derived Compounds, Kaempferol and Hydroxysafflor Yellow A, on Hyperglycaemic Stress-Induced Podocyte Apoptosis via Modulating of Macrophage M1/M2 Polarization

**DOI:** 10.1155/2020/2462039

**Published:** 2020-10-10

**Authors:** Yuanping Li, Dan Zheng, Dayue Shen, Xilan Zhang, Xiaoyun Zhao, Hui Liao

**Affiliations:** ^1^Department of Pharmacy, Shanxi Provincial People's Hospital of Shanxi Medical University, Taiyuan 030012, China; ^2^School of Pharmacy, Shanxi Medical University, Taiyuan 030001, China

## Abstract

**Objective:**

The primary initiating mechanism in diabetes nephropathy (DN) is hyperglycemia-induced inflammation in which macrophage and podocyte play important roles. The present research is aimed at exploring the effects of kaempferol (Ka) and hydroxysafflor yellow A (HSYA) on classically activated (M1)/alternatively activated (M2) macrophage polarization and podocyte apoptosis under hyperglycaemic conditions *in vitro*.

**Methods:**

(1) RAW264.7 cells were treated with 11.1 mM glucose (NG), 33.3 mM glucose (HG), Ka 4–8 *μ*M, and HSYA 100–200 *μ*M separately. The expressions of inducible nitric oxide synthase (iNOS), tumor necrosis factor- (TNF-) *α*, mannose receptor (CD206), and arginase- (Arg-) 1 were quantified by Western blotting and real-time quantitative PCR. The collected supernatants from macrophage were named as (NG) MS, (HG) MS, (Ka) MS, and (HSYA) MS. (2) The podocyte survival rate was assessed by Bromodeoxyuridine assay, while TNF-*α* and interleukin- (IL-) 1*β* levels were evaluated by Elisa.

**Results:**

(1) Compared to the HG group, the Ka and HSYA 100 *μ*M groups decreased iNOS and TNF-*α* levels and increased Arg-1 and CD206 expressions significantly (protein and mRNA: *p* < 0.05, respectively). (2) The podocyte survival rate of Ka 8 *μ*M was higher than that of HG, and the rates of (Ka) MS and (HSYA 100 *μ*M) MS were higher than that of (HG) MS significantly (all: *p* < 0.05). (3) TNF-*α* and IL-1*β* levels of Ka and HSYA 100 *μ*M were significantly lower than those of the HG group, and both levels in the (Ka) MS and (HSYA) MS were lower than those in the (HG) MS group significantly (*p* < 0.05, respectively).

**Conclusion:**

The protective effects of Ka and HSYA on podocyte apoptosis under hyperglycemic stress are related to their modulation on M1/M2 polarization and the lowering effects on TNF-*α* and IL-1*β* levels.

## 1. Introduction

Research data from China and the United States showed that the prevalence of chronic kidney disease (CKD) in adults is about 13% [1, 2]. A recent study indicated that diabetes nephropathy (DN) is currently the leading cause of CKD in China [3]. Traditional Chinese medicine (TCM) has already shown its effects in treating DN proteinuria and was able to provide a variety of effective renoprotective extracts [4]. In recent years, kaempferol (Ka) and hydroxysafflor yellow A (HSYA), two active components of safflower, attracted attention for their roles in ameliorating diabetes-induced fibrosis and renal damage [5, 6]. Safflower yellow injection (SYI), which contains more than 90% HSYA, is recommended by TCM experts for clinical treatment of DN in China [7].

From the perspective of pathophysiology, both macrophage-mediated inflammation [8] and then inflammation-induced podocyte apoptosis [9] play significant roles in the development and progression of DN. It is reported that hyperglycemia is a major driving force in activated-macrophages infiltration in the kidney and promotes podocyte apoptosis [10]. In addition, classically activated (M1) macrophages, but not alternatively activated (M2) macrophages, induced podocyte permeability [11]. However, the protective effects of HSYA and Ka on podocyte apoptosis related to M1/M2 macrophage-mediated mechanism are rarely mentioned.

Our previous study indicated that the safflower extracts could inhibit lipopolysaccharide- (LPS-) induced M1 activation and decrease tumor necrosis factor- (TNF-) *α* and interleukin- (IL-) 1*β* levels in RAW264.7 cells [12]. In the present study, we investigated the effects of Ka and HSYA on the expressions of two markers of M1 polarization, TNF-*α* and inducible nitric oxide synthase (iNOS), and two markers of M2 polarization, mannose receptor (CD206) and arginase- (Arg-) 1 under hyperglycemic conditions. Additionally, we further evaluated the effects of Ka and HSYA on hyperglycemic stress-induced podocyte apoptosis via M1/M2 polarization, and the contents of TNF-*α* and IL-1*β* in podocyte.

## 2. Methods

### 2.1. Cell Culture

A mouse RAW264.7 macrophage cell line was purchased from Absin Biotechnology Company (Shanghai, China) and cultured in Roswell park memorial institute medium (RPMI-1640, HyClone, GE Healthcare Life Sciences, Logan, UT, USA) supplemented with 10% fetal bovine serum (FBS, Gibco BRL, Gaithersburg, MD, USA) in a 5% CO_2_ incubator at 37°C. The medium was replaced the next day. The serum-free RPMI-1640 medium was synchronized for 12 hours before the intervention.

A conditionally immortalized mouse MPC-5 podocyte cell line was purchased from Fuheng Biology Company (Shanghai, China) and cultured in an RPMI-1640 medium supplemented with 10% FBS and recombinant IFN-*γ* (G1021, APExBIO, USA) at 33°C. Podocytes were reseeded and cultured in an RPMI-1640 medium with 11.1 mM glucose and 10 mg/ml type-I collagen (BD Bioscience, Bedford, MA, USA) at 37°C without IFN-*γ* for 7–15 days to induce differentiation.

### 2.2. Determination of Hyperglycemic Conditions Capable of Inducing Phenotypic Transition of M1 Macrophage

RAW 264.7 cells (99 *μ*l, plated at 1 × 10^6^ cells/ml) were stimulated with LPS (1 *μ*l, 0.5 *μ*g/ml, Wako Chemicals USA Inc., Richmond, VA, USA), and the solvent control dimethyl sulfoxide (DMSO), as well as 5.6 mM, 11.1 mM, 25.0 mM, 33.3 mM, and 44.4 mM glucose, respectively, for 24 hours and 48 hours, before assessment of nitric oxide (NO) production. LPS stimulated cells were cultured in a normal medium and considered as the model control [13]. The 5.6 mM and the 11.1 mM glucose groups were considered as the normal glucose groups [14, 15]. The 25.0 mM–44.4 mM glucose groups were referred to as the high glucose concentration groups [15, 16]. Nitrite, a stable end-product of NO metabolism, was measured using the Griess reaction [17].

### 2.3. Determination of Safe Concentration Levels of Ka and HSYA in Macrophages under Hyperglycemic Conditions

Macrophages were separately seeded into 96-well plates at a density of 5 × 10^4^ cells/ml and cultured in a 10% FBS RPMI-1640 medium for 24 hours. Following another 24-hour treatment with 11.1 mM glucose and 33.3 mM glucose (above NG and HG concentrations were based on the results from Method 2.2), Ka (National Institutes for Food and Drug Control, ≥ 98%) at 4, 8, and 12 *μ*M [18], and HSYA (National Institutes for Food and Drug Control, ≥ 99%) at 100, 200, and 300 *μ*M [19] were added into HG. The supernatants were removed, and each well washed with PBS before the addition of 10% FBS RPMI-1640 medium and 10 *μ*l CCK-8 reagent (Boster Biological Technology, Wuhan, China). Cell viability was determined by measuring the absorbance at 450 nm using a microporous plate reader (Model 550; Bio-Rad Laboratories, Inc., Hercules, CA, USA) after an incubation period of 2 hours at 37°C. The average optical density was determined by examining six wells per group.

### 2.4. The Effects of Ka and HSYA on iNOS, TNF-*α*, Arg-1, and CD206 Protein Expressions in HG-Induced Macrophages as Measured by Western Blotting [12]

The iNOS, TNF-*α*, Arg-1, and CD206 protein expressions at different concentrations of Ka (4 *μ*M, 8 *μ*M) and HSYA (100 *μ*M, 200 *μ*M) in HG-cultured macrophages were tested by Western blotting. The treated cells (1 × 10^6^ cells/ml) were removed from the culture media and lysed with RIPA lysis buffer from Solarbio Science & Technology (Beijing, China) for 30 minutes. The protein concentrations were determined using a BCA Protein Assay Kit from Boster Biological Technology (Wuhan, China). Samples containing 50 *μ*g of protein were resolved by 12% SDS-PAGE electrophoresis and transferred to nitrocellulose membranes (Solarbio Science & Technology, Beijing, China) in a buffer tank with platinum wire electrodes. Nonspecific binding was blocked by immersing the membranes into 5% nonfat dried milk (diluted in 0.1% (*v*/*v*) Tween-20 PBS) for 3 hours at room temperature. After rinsing several times with a washing buffer (0.1% Tween-20 in PBS), the membranes were incubated overnight with a primary antibody against iNOS at 1 : 500 dilution (Catalog No. BA0362, Boster), a primary antibody against TNF-*α* (Catalog No. BA0131, Boster) at 1 : 500 dilution, a primary antibody against CD206 (Catalog No. A02285-2, Boster) at 1 : 500 dilution, and a primary antibody against Arg-1 (Catalog No. BM4000, Boster) at 1 : 500 dilution at 4°C. The membranes were then washed three times (10 minutes each), then incubated with the corresponding secondary IgG conjugated to HRP antibody (Proteintech, Wuhan, China) at room temperature for 3 hours. The results were finally analyzed by the Quantity One analysis system (Bio-Rad, Hercules, CA, USA). GAPDH at a dilution of 1 : 1000 (Catalog No. A00227-1, Boster) was used as the internal loading control.

### 2.5. The Effects of Ka and HSYA on iNOS, TNF-*α*, Arg-1, and CD206 Expressions in HG-Induced Macrophages as Measured by Real-Time Quantitative PCR [12]

Total RNAs were extracted from NG-treated RAW 264.7 cells, HG-activated cells, and sample-treated cells (1 × 10^6^ cells/ml) by Trizol Reagent (Ambion, USA). An equal amount (1 *μ*g) of RNAs were reverse transcribed using a high capacity RNA-to-cDNA PCR kit (Takara, Beijing, China). Mouse gene PCR primer sets for iNOS, TNF-*α*, Arg-1, and CD206 were obtained from SABiosciences (Germantown, MD). The Power SYBR Green PCR Master Mix (Applied Biosystems) was used with the step-one-plus real-time PCR system (Applied Biosystems). The protocol included denaturing for 15 min at 95°C, 40 cycles of three-step PCR including denaturing for 15 sec at 95°C, annealing for 30 sec at 58°C, and extension for 30 sec at 72°C, with an additional 15-second detection step at 81°C, followed by a melting profile from 55°C to 95°C at a rate of 0.5°C per 10 sec. The samples of 25 ng cDNA were analyzed in quadruplicate in parallel with ribosomal protein lateral stalk subunit p1 (RPLP1)/3 controls. Standard curves (threshold 1 cycle vs. log 2 pg cDNA) were generated from a series of log dilutions of standard cDNA (reverse transcribed from mRNA from RAW264.7 cells in growth media) from 0.1 pg to 100 ng. Initial quantities of experimental mRNA were then calculated from the standard curves and averaged using the SA Bioscience software. The ratio of the experimental four marker genes to RPLP1/3 mRNA was calculated and normalized to NG.

### 2.6. Collection of the Supernatant from Macrophages [20]

RAW264.7 cells were seeded into a six-well plate and cultured in NG, HG, Ka 4, 8 *μ*M in HG and HSYA 100, and 200 *μ*M in HG, respectively. The supernatants were collected 24 hours later, centrifuged at 1,500 g for 15 minutes, and labeled as (NG) MS, (HG) MS, (Ka4) MS, (Ka8) MS, (HSYA100) MS, and (HSYA200) MS. The collected supernatants were centrifuged for the second time at 1,500 g for 10 minutes at 4°C, filtered with 0.22 *μ*m sterile membrane, and stored at -80°C for further use.

### 2.7. Determination of TNF-*α* and IL-1*β* Levels in Podocytes

Podocytes (5 × 10^5^ cells/ml) were treated with HG, Ka (4 and 8 *μ*M), HSYA (100 and 200 *μ*M), (NG) MS, (HG) MS, (Ka4) MS, (Ka8) MS, (HSYA100) MS, and (HSYA200) MS separately. NG was designated as the normal control for all the above-tested groups. Cell supernatants were then harvested and centrifuged at 1,500 g for 10 minutes at 4°C after 24 hours. TNF-*α* and IL-1*β* levels were determined using an ELISA kit (Catalog No. EK0527 and EK0394, Boster). The absorbance was measured using a microplate reader (Model 550; Bio-Rad Laboratories, Inc.). Each sample underwent repeated testing (six times).

### 2.8. Determination of Podocytes Survival Rate Using Bromodeoxyuridine (BrdU) Assay [21]

Before stimulation, podocytes (5 × 10^5^ cells/ml) were cultured overnight with serum-starvation then treated with NG, HG, Ka 4 *μ*M, Ka 8 *μ*M, HSYA 100 *μ*M, HSYA 200 *μ*M, (NG) MS, (HG) MS, (Ka4) MS, (Ka8) MS, (HSYA100) MS, and (HSYA200) MS for 22 hours. Podocytes were then seeded into 96-well plates. The cells were subsequently treated with BrdU during the final 2 hours of incubation. The BrdU cell proliferation assay kit (cat. no. 2750; EMD Millipore, Billerica, MA, USA) was performed according to the manufacturer's protocol. The NG group was used as the normal control to determine cell mortality. The average was obtained by examining six wells per group.

### 2.9. Statistical Analysis

The SPSS 19.0 software (IBM, Armonk, NY, US) was used for statistical analysis. All the data were expressed as mean ± standard error of the mean. The among group comparisons were conducted by one-way analysis of variance followed by Dunnett's multiple comparison tests for continuous variables. All the reported *p* values were two-tailed, and a *p* < 0.05 was considered as statistically significant.

## 3. Results

### 3.1. Determination of Hyperglycemic Conditions Capable of Inducing Phenotypic Transition of M1 Macrophage

Different concentrations of glucose were tested by CCK-8 assay to study their impact on RAW264.7 cell viability before the evaluation of NO production. The results did not indicate a significant difference in cell survival rates between the 5.6, 11.1, 25.0, 33.3, and 44.4 mM glucose concentrations (data not shown).

**Figure 1:**
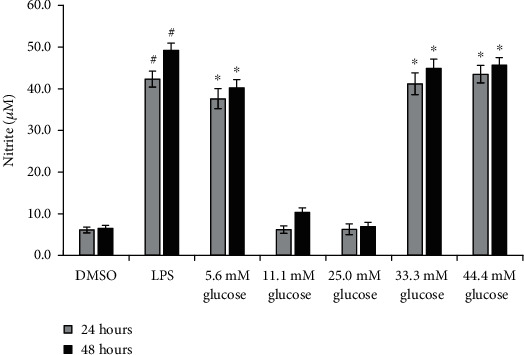
Determination of hyperglycemic conditions capable of inducing phenotypic transition of M1 macrophage. Values were expressed as mean ± standard error of the mean (*n* = 6). ^#^*p* < 0.05 versus DMSO after 24 hours' and 48 hours' treatment separately. ^∗^*p* < 0.05 versus 11.1 mM glucose after 24 hours' and 48 hours' treatment separately. Abbreviations: LPS: lipopolysaccharide; DMSO: dimethyl sulfoxide, the solvent control of LPS.

The analysis results indicated that the 24 and 48 hours LPS-induced NO productions were significantly higher than those of DMSO (the solvent control of LPS) (*p* < 0.001, Figure 1). We proceeded to explore the hyperglycemic conditions in this study, taking 0.5 *μ*g/ml LPS as the M1 model control [18].

Significant differences were seen between the 24-hour and 48-hour incubation periods NO productions of macrophages in the 5.6 mM glucose group and the NG group (both: *p* < 0.001). Meanwhile, no significant difference in NO production was seen between the NG and DMSO groups at 24-hour time point (*p* = NS).

The results also showed that the 24- and 48-hour incubation period macrophages in the HG and 44.4 mM glucose groups had significantly higher nitrite levels as compared to the NG group (all: *p* < 0.01). No statistically significant difference in LPS-induced NO production was seen between the HG and 44.4 mM glucose groups 24 hours and 48 hours after the treatment (all: *p* = NS).

Based on the above results, the HG group and the 24 hours after treatment time point were used as standard hyperglycemic conditions for M1 polarization, while the NG groups were used as control groups for HG.

### 3.2. Determination of Safety Concentrations of Ka and HSYA in Macrophages under Hyperglycemic Conditions

**Figure 2:**
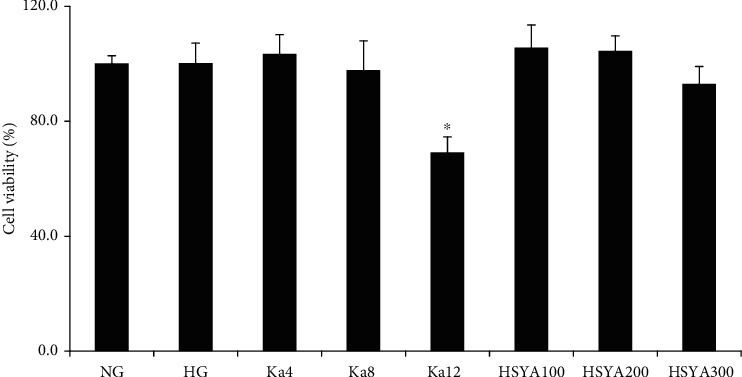
Determination of safety concentrations of Ka and HSYA in macrophages under hyperglycemic conditions with CCK-8 assay. ^∗^*p* < 0.05 versus NG. Abbreviations: NG: 11.1 mM glucose; HG: 33.3 mM glucose; Ka4: kaempferol at 4 *μ*M; Ka8: kaempferol at 8 *μ*M; Ka12: kaempferol at 12 *μ*M; HSYA100: hydroxysafflor yellow A at 100 *μ*M; HSYA200: hydroxysafflor yellow A at 200 *μ*M; HSYA300: hydroxysafflor yellow A at 300 *μ*M.

Figure 2 shows that no significant difference in macrophage survival rate was observed between the HG and the NG groups (*p* = NS). The results also showed that HSYA concentrations of 100, 200, and 300 *μ*M, as well as Ka concentrations of 4 *μ*M and 8 *μ*M, had no significant influence on cell survival rate compared to NG-treated macrophages (all: *p* = NS).

### 3.3. The Effects of Ka and HSYA on iNOS, TNF-*α*, Arg-1, and CD206 Protein Levels and mRNA Expressions in HG-Induced Macrophages

**Figure 3:**
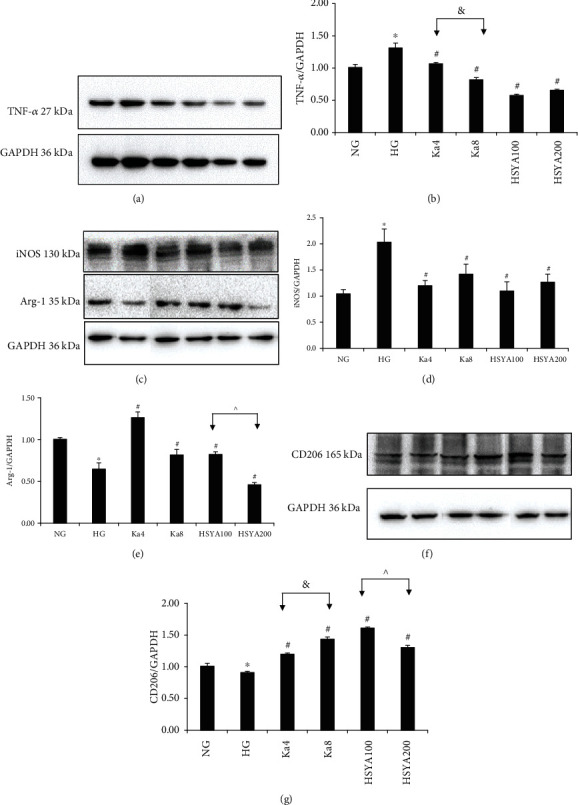
The effects of Ka and HSYA on iNOS, TNF-*α*, Arg-1, and CD206 protein expressions in HG-induced macrophages as measured by Western blotting. TNF-*α* protein expression was shown in (a) and represented in (b). iNOS and Arg-1 protein expressions were shown in (c) and represented in (d, e). CD206 protein expression was shown in (f) and represented in (g). All results were expressed as a ration with respect to control and represented as the mean ± SD in triplicates. ^∗^*p* < 0.05 versus NG. ^#^*p* < 0.05 versus HG. ^&^*p* < 0.05, Ka4 versus Ka8. ^^^*p* < 0.05, HSYA100 versus HSYA200. Abbreviations: NG: 11.1 mM glucose; HG: 33.3 mM glucose; Ka4: kaempferol 4 *μ*M in HG; Ka8: kaempferol 8 *μ*M in HG; HSYA100: hydroxysafflor yellow A 100 *μ*M in HG; HSYA200: hydroxysafflor yellow A 200 *μ*M in HG; TNF-*α*: tumor necrosis factor-*α*; iNOS: inducible nitric oxide synthase; CD206: mannose receptor; Arg-1: arginase-1.

Our results showed that compared to the NG group, the HG group had significantly higher TNF-*α* and iNOS protein expressions (Figures 3(a)–3(d): *p* < 0.05) as well as significantly lower Arg-1 and CD206 expressions (Figures 3(c), 3(e), 3(f), and 3(g): *p* < 0.05).

**Figure 4:**
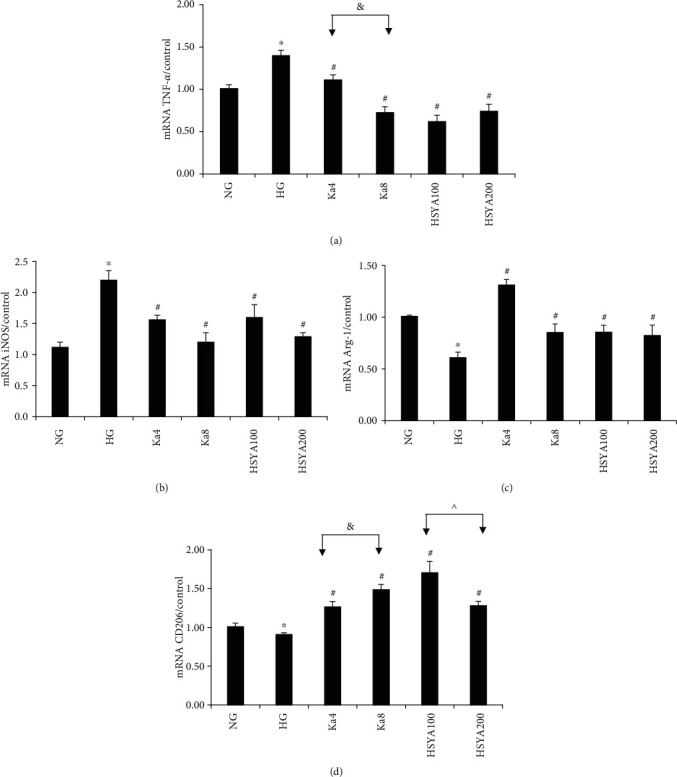
The effects of Ka and HSYA on iNOS, TNF-*α*, Arg-1, and CD206 expressions in HG-induced macrophages as measured by real-time quantitative PCR. (a) TNF-*α* results. (b) iNOS results. (c) Arg-1 results. (d) CD206 results. All results were expressed as a ration with respect to control and represented as the mean ± SD in triplicates. ^∗^*p* < 0.05 versus NG. ^#^*p* < 0.05 versus HG. ^&^*p* < 0.05, Ka4 versus Ka8. ^^^*p* < 0.05, HSYA100 versus HSYA200. Abbreviations: NG: 11.1 mM glucose; HG: 33.3 mM glucose; Ka4: kaempferol 4 *μ*M in HG; Ka8: kaempferol 8 *μ*M in HG; HSYA100: hydroxysafflor yellow A 100 *μ*M in HG; HSYA200: hydroxysafflor yellow A 200 *μ*M in HG; TNF-*α*: tumor necrosis factor-*α*; iNOS: inducible nitric oxide synthase; CD206: mannose receptor; Arg-1: arginase-1.

The mRNA expression results were similar to those of protein expressions: TNF-*α* and iNOS mRNA increased in the HG-treated group (Figures 4(a) and 4(b)), while Arg-1 and CD206 decreased in the same group (Figures 4(c) and 4(d)), and all showed significant differences when compared to those of the NG group (all: *p* < 0.05).

Compared to HG, Ka 4–8 *μ*M and HSYA 100–200 *μ*M not only significantly suppressed iNOS and TNF-*α* protein expressions but also iNOS and TNF-*α* mRNA expressions (all: *p* < 0.05).

Additionally, the results showed that the Ka 4–8 *μ*M and HSYA 100 *μ*M groups had significantly increased Arg-1 protein level and mRNA expression (all: *p* < 0.01) as compared to HG. Meanwhile, compared to the HG group, the Ka 4–8 *μ*M and HSYA 100–200 *μ*M groups also showed a significant increase in CD206 protein levels and mRNA expressions (all: *p* < 0.05).

Significant dose-dependent effects of various concentrations of Ka (4–8 *μ*M) can be seen on TNF-*α* protein expression (*p* = 0.001), TNF-*α* mRNA expression (*p* = 0.002), as well as CD206 protein (*p* = 0.001), and CD206 mRNA expressions (*p* = 0.015).

The HSYA 100 *μ*M group showed far better improvements in Arg-1 and CD206 expressions (Arg-1 protein: *p* < 0.001, CD206 protein: *p* = 0.005, and CD206 mRNA: *p* = 0.024) compared to the HSYA 200 *μ*M group.

It was interesting to see that the HSYA 200 *μ*M group exhibited conflicting effects on Arg-1 protein level and mRNA expression (it significantly decreased Arg-1 protein level (*p* = 0.036), but significantly increased Arg-1 mRNA expression (*p* = 0.046)).

### 3.4. Determination of TNF-*α* and IL-1*β* Levels in Podocytes

Compared to the NG group, both TNF-*α* and IL-1*β* expressions increased significantly after HG stimulation (TNF-*α*: (76.9 ± 1.6) pg/ml vs. (21.1 ± 2.6) pg/ml, *p* < 0.001. IL-1*β*: (37.8 ± 2.9) pg/ml vs. (17.9 ± 1.3) pg/ml, *p* < 0.001). Compared to HG, (HG) MS significantly promoted TNF-*α* and IL-1*β* level to (116.7 ± 9.6) pg/ml and (64.1 ± 3.2) pg/ml (*p* < 0.001).

**Figure 5:**
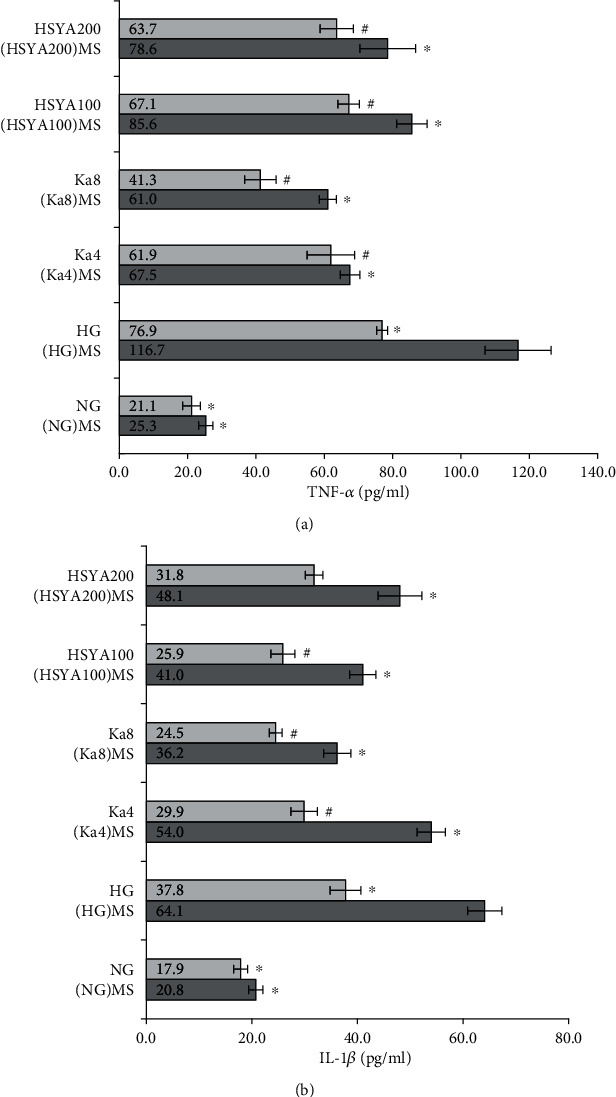
Determination of TNF-*α* and IL-1*β* levels in podocytes. (a) TNF-*α* level. (b) IL-1*β* level. Values were expressed as mean ± standard error of the mean (*n* = 6). ^∗^*p* < 0.05 versus (HG)MS. ^#^*p* < 0.05 versus HG. Abbreviations: TNF-*α*: tumor necrosis factor-*α*; IL-1*β*: interleukin-1*β*; NG: 11.1 mM glucose; HG: 33.3 mM glucose; Ka4: kaempferol 4 *μ*M in HG; Ka8: kaempferol 8 *μ*M in HG; HSYA100: hydroxysafflor yellow A 100 *μ*M in HG; HSYA200: hydroxysafflor yellow A 200 *μ*M in HG; (NG) MS: the supernatants from NG-cultured macrophage; (HG) MS: the supernatants from HG-cultured macrophage; (Ka4) MS: the supernatants from kaempferol 4 *μ*M and HG-cultured macrophage: (Ka8) MS: the supernatants from kaempferol 8 *μ*M and HG-cultured macrophage; (HSYA100) MS: the supernatants from hydroxysafflor yellow A 100 *μ*M and HG-cultured macrophage; (HSYA200) MS: the supernatants from hydroxysafflor yellow A 200 *μ*M and HG-cultured macrophage.

All Ka 4 *μ*M, Ka 8 *μ*M, HSYA 100 *μ*M, and HSYA 200 *μ*M groups had significantly lower TNF-*α* and IL-1*β* levels as compared to the HG group (all: *p* < 0.05). Additionally, the results of (Ka4) MS, (Ka8) MS, (HSYA100) MS, and (HSYA200) MS groups also showed a significant decrease in TNF-*α* and IL-1*β* levels as compared to the (HG) MS group (all: *p* < 0.01). All those results are shown in Figure 5.

### 3.5. Determination of Cell Survival Rate of Podocytes

**Figure 6:**
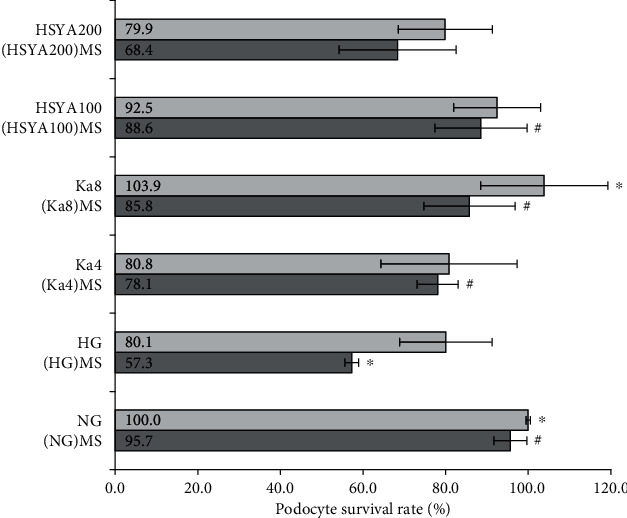
Determination of cell survival rate of podocytes. Values were expressed as mean ± standard error of the mean (*n* = 6). ^∗^*p* < 0.05 versus HG. ^#^*p* < 0.05 versus (HG) MS. Abbreviations: NG: 11.1 mM glucose; HG: 33.3 mM glucose; Ka4: kaempferol 4 *μ*M in HG; Ka8: kaempferol 8 *μ*M in HG; HSYA100: hydroxysafflor yellow A 100 *μ*M in HG; HSYA200: hydroxysafflor yellow A 200 *μ*M in HG; (NG) MS: the supernatants from NG-cultured macrophage; (HG) MS: the supernatants from HG-cultured macrophage; (Ka4) MS: the supernatants from kaempferol 4 *μ*M and HG-cultured macrophage; (Ka8) MS: the supernatants from kaempferol 8 *μ*M and HG-cultured macrophage; (HSYA100) MS: the supernatants from hydroxysafflor yellow A 100 *μ*M and HG-cultured macrophage; (HSYA200) MS: the supernatants from hydroxysafflor yellow A 200 *μ*M and HG-cultured macrophage.

From Figure 6, we can see that compared to the NG group, the cell survival rate in HG treated cells decreased significantly ((80.1 ± 11.2)% vs. (100.0 ± 0.5)%: *p* = 0.016). The (HG) MS group had the lowest survival rate ((57.3 ± 1.7)%) among all tested groups, and the difference was statistically significant when compared with the HG group (*p* < 0.001).

Ka 8 *μ*M significantly increased the cell survival rate compared to the HG group ((103.9 ± 15.4)%, *p* = 0.025). However, HSYA did not show a direct influence on the survival rate of podocytes in HG.

All (Ka4) MS, (Ka8) MS, and (HSYA100) MS groups exhibited significant protective effects on podocyte apoptosis compared to the (HG) MS group (*p* < 0.05).

## 4. Discussion

Since 1978 when for the first time a combination of diabetes and blood stasis syndrome was proposed by an expert of TCM Dr. Chenyu Zhu, much progress has been made in the treatment of diabetes and its complications with blood-activating and stasis-resolving herbs [22]. Safflower, the tubular flower of *Carthamus tinctorius*, has been shown to promote blood circulation, remove blood stasis, and has been used to treat conditions such as diabetes-related cardiovascular diseases and diabetes-related CKD [23, 24]. From a modern pharmacological perspective, the activity of safflower is related to its main compounds: Ka and HSYA [25, 26].

The primary initiating mechanism in DN is hyperglycemia-induced vascular dysfunction. However, its progression is due to different pathological mechanisms, including oxidative stress and inflammatory cell infiltration [27]. The growing evidence indicates that immunological and inflammatory mechanisms play an important role in the development and progression of DN [28]. There are two major sources of inflammatory cells involved in the inflammation process: first, bone marrow-derived leukocytes that include macrophages and mast cells; second, locally activated kidney cells such as mesangial cells and podocytes [29].

The specific glucose concentration able to induce macrophage M1/M2 polarization was determined before sampling and testing. According to current references, macrophage polarization can be induced by high glucose concentrations ranging from 25.0 mM to 45.0 mM [15, 16]. Meanwhile, 5.6 mM glucose and NG are normally regarded as normal glucose controls in different cell lines [14, 15]. It is interesting to see that 5.6 mM glucose significantly induced higher NO production than NG in RAW264.7 cells. Further, Western blot analysis confirmed that the 5.6 mM glucose group yielded significantly higher iNOS protein expressions than the NG group after 24 hours of treatment (data not shown). This result provided further ideas on the effect comparison of Ka and HSYA in 5.6 mM glucose and HG-induced macrophage polarization for our additional research.

Among the four tested proteins, we firstly paid close attention to TNF-*α* expression. Treatment of ischemia/reperfusion rats with HSYA markedly reduced the levels of blood urea nitrogen, attenuated renal cell apoptosis, and reduced IL-1*β* and TNF-*α* release [30]. iNOS is another important indicator of macrophage polarized to the M1 subtype [31]. Inhibitory effects of Ka and HSYA on iNOS expressions have also been previously reported [32, 33]. Our present study further confirmed that both Ka and HSYA have inhibitory effects on HG-induced TNF-*α* and iNOS.

Additionally, the expressions of CD206 and Arg-1 were upregulated when Ka and HSYA were used on HG-treated cells. This is to our knowledge the first study to explore the effects of Ka and HSYA on M2 polarization. Several previous studies have established that the increased expressions of Arg-1 and CD206 are indicators of M2 polarization [34, 35]. A targeted intervention on M1/M2 macrophage polarization might be a novel therapeutic strategy for the treatment of DN [36]. The identification of the effects of Ka and HSYA on the M2 subtype might provide new insights and a basis for macrophage-related therapeutic strategies for DN.

A previous study has revealed that both increased M1 and decreased M2 play a crucial role in podocyte injury *in vivo* [37]. A series of cell experiments in isolated culture or the coculture of macrophages and podocytes was conducted to examine their interaction [20, 38, 39]. Our results showed that (HG) MS, the isolated culture of HG-treated macrophage, induced podocyte apoptosis. Additionally, podocyte apoptosis is more severe in (HG) MS than in HG.

A mechanism study demonstrated that activated macrophages could induce podocyte injury via a TNF-*α*-dependent pathway [40]. Apart from TNF-*α*, many inflammatory factors, including IL-1*β* and IL-6, take part in the inflammatory process and contribute to podocyte injury [41]. Our research confirmed that the increase in TNF-*α* and IL-1*β* levels in podocyte played an important role in HG and (HG) MS-induced podocyte apoptosis. Ka showed direct protective effects on podocytes, and both Ka and HSYA exerted indirect effects on podocyte under hyperglycemic conditions through modulation of M1/M2 polarization and reduction of TNF-*α* and IL-1*β* levels.

It is reported that the coculture of macrophage-derived extracellular vesicles (EVs) and podocytes promoted reactive oxygen species (ROS) production and activation of inflammatory in podocyte line [42]. Additionally, infiltrating macrophages in DN promote podocyte apoptosis via the TNF-*α*-ROS pathway [10]. As an important mediator for the activation of proinflammatory signaling pathways, ROS production may favor the induction of M1-like proinflammatory macrophages and block the polarization of M2 anti-inflammatory macrophages during the onset and progression of diabetes [43, 44]. Therefore, a comprehensive understanding of the impact between ROS and coculture of macrophage and podocyte apoptosis under hyperglycemic stress is needed.

As we mentioned before, SYI is recommended in the treatment of renal disease such as DN, hematodialysis [7]. Multiple clinical studies support the above recommendation: many randomized controlled trials (RCT) showed that routine treatment plus SYI can significantly inhibit the inflammatory response and reduce urinary protein in DN patients [45]. SYI combined with benazepril is better than benazepril alone in improving renal function and hypercoagulability of DN patients [46]. A RCT study involving 70 hemodialysis patients showed that SYI can improve the prognosis of patients [47]. Other studies have shown that SYI can also improve insulin resistance and correct lipid metabolism disorder in nondiabetic hemodialysis patients [48]. On the other hand, the clinical application of HSYA in DN has been promoted by the pharmacokinetic properties and the safety experiment in healthy Chinese volunteers [7, 49].

Unlike HSYA, which only exists in safflower, kaempferol is one of the most ubiquitous polyphenols in fruits and vegetables [50]. Our research showed that the concentrations of Ka acting on macrophage and podocyte were not only lower than HSYA but also showed both direct and indirect protection on podocyte apoptosis under hyperglycaemic conditions. Considering the results of recent clinical researches on rheumatoid arthritis, the possibility of a transition of Ka from chemistry to clinical medicine becomes even greater [51]. However, due to a lack of clinical trials, there is still a long way to go for a successful recommendation of Ka as novel therapeutic options for DN patients.

Summary of our results: (1) Ka and HSYA exhibited modulatory effects on the M1/M2 phenotype under hyperglycaemic conditions. (2) Ka and HSYA displayed indirect protective effects on podocyte apoptosis through the modulation of macrophage M1/M2 differentiation. (3) The clinical application of HSYA in the treatment of DN has made great progress in China. (4) Ka also showed a direct protection on podocyte apoptosis under hyperglycaemic conditions. We look forward to its clinical application in the future.

## Data Availability

All authors declare that the data of the manuscripts can be verified the results of the article, be replicated the analysis, and be conducted secondary analyses. The readers can access the data supporting the conclusions of the study within the article.
